# Acceptability of COVID-19 vaccine by paramedics

**DOI:** 10.1192/j.eurpsy.2021.814

**Published:** 2021-08-13

**Authors:** H. El Kefi, K. Kefi, W. Krir, C. Bencheikh Brahim, A. Baatout, I. Bouzouita, H. Slema, A. Oumaya

**Affiliations:** Psychiatric Unit, Military Hospital of Tunis, tunis, Tunisia

**Keywords:** Vaccination, Vaccination Refusal, Immunization, coronavirus

## Abstract

**Introduction:**

The year 2020 was marked by the COVID-19 pandemic that killed more than one million people. Scientists around the world are looking for an effective vaccine against this virus.

**Objectives:**

The objective of our study was to assess the acceptability of the COVID-19 vaccine by paramedics.

**Methods:**

Descriptive and cross-sectional study including paramedics (nurses, orderlies) from the military hospital of Tunis. Data collection was carried out by a clinical psychologist. We studied the associations between the different characteristics of our population and the decision to accept or refuse vaccination against COVID-19.

**Results:**

A total of 161 paramedics agreed to answer our questionnaire. The average age was 37.73 years. The average number of years worked was 14.95 years. There were 85 women (52.8%) and 76 men (47.2%). The rapid discovery of the vaccine was hoped for by 94.4%. Vaccination was considered a means of collective protection by 84.5%. However, only 52.8% agreed to be vaccinated by the COVID-19 vaccine. The main factors significantly associated with refusal of the COVID-19 vaccine were previous refusal of influenza vaccination (p = 0.006).

**Conclusions:**

Apprehension about vaccination does not appear to be sparing the future COVID-19 vaccine. To achieve vaccination coverage that would protect health care workers, several awareness and communication activities must be carried out.

